# Measuring childhood maltreatment to predict early-adult psychopathology: Comparison of prospective informant-reports and retrospective self-reports

**DOI:** 10.1016/j.jpsychires.2017.09.020

**Published:** 2018-01

**Authors:** Joanne B. Newbury, Louise Arseneault, Terrie E. Moffitt, Avshalom Caspi, Andrea Danese, Jessie R. Baldwin, Helen L. Fisher

**Affiliations:** a MRC Social, Genetic & Developmental Psychiatry Centre, Institute of Psychiatry, Psychology & Neuroscience, King's College London, London, UK; b Department of Psychology and Neuroscience, Duke University, Durham, NC, USA; c Department of Psychiatry and Behavioral Sciences, Duke University Medical School, Durham, NC, USA; d Department of Child & Adolescent Psychiatry, Institute of Psychiatry, Psychology & Neuroscience, King's College London, London, UK; e National and Specialist CAMHS Trauma and Anxiety Clinic, South London and Maudsley NHS Foundation Trust, London, UK

**Keywords:** Child abuse, Mental health, Adolescence, Early adulthood, Assessment, Recall bias

## Abstract

Both prospective informant-reports and retrospective self-reports may be used to measure childhood maltreatment, though both methods entail potential limitations such as underestimation and memory biases. The validity and utility of standard measures of childhood maltreatment requires clarification in order to inform the design of future studies investigating the mental health consequences of maltreatment. The present study assessed agreement between prospective informant-reports and retrospective self-reports of childhood maltreatment, as well as the comparative utility of both reports for predicting a range of psychiatric problems at age 18. Data were obtained from the Environmental Risk (E-Risk) Longitudinal Twin Study, a nationally-representative birth cohort of 2232 children followed to 18 years of age (with 93% retention). Childhood maltreatment was assessed in two ways: (i) prospective informant-reports from caregivers, researchers, and clinicians when children were aged 5, 7, 10 and 12; and (ii) retrospective self-reports of maltreatment experiences occurring up to age 12, obtained at age 18 using the Childhood Trauma Questionnaire. Participants were privately interviewed at age 18 concerning several psychiatric problems including depression, anxiety, self-injury, alcohol/cannabis dependence, and conduct disorder. There was only slight to fair agreement between prospective and retrospective reports of childhood maltreatment (all Kappa's ≤ 0.31). Both prospective and retrospective reports of maltreatment were associated with age-18 psychiatric problems, though the strongest associations were found when maltreatment was retrospectively self-reported. These findings indicate that prospective and retrospective reports of childhood maltreatment capture largely non-overlapping groups of individuals. Young adults who recall being maltreated have a particularly elevated risk for psychopathology.

## 1 Background

The 10% of children affected by maltreatment worldwide (Butchart et al., 2006, Gilbert et al., 2009, Radford et al., 2011, Radford et al., 2013) shoulder a disproportionate burden of mental illness. Abused and neglected children experience more concurrent psychiatric problems (Arseneault et al., 2011, Cicchetti and Toth, 2005, Jaffee et al., 2004), and the deleterious sequelae of childhood maltreatment appear to extend across the life span (Green et al., 2010, Kessler et al., 2010, MacMillan et al., 2001), including debilitating adult conditions such as depression (Widom et al., 2007a), alcohol abuse (Widom et al., 2007b), psychosis (Varese et al., 2012), and suicidal behavior (Ystgaard et al., 2004). The emotional and economic implications of severe mental illness are considerable (Vigo et al., 2016), and there are now international calls to understand the health toxicity and potential reversibility of adverse childhood experiences like maltreatment (Butchart et al., 2006, National Institute of Aging, 2012, National Scientific Council on the Developing Child, 2007). A fundamental step in this health initiative is to examine the validity and utility of standard measures of maltreatment, in order to understand the basis of the association between childhood maltreatment and adult psychopathology and inform future research.

Most evidence linking childhood maltreatment with adult mental health problems comes from cross-sectional studies of adults who retrospectively report on their childhood experiences (Hardt and Rutter, 2004). Because these self-reports are usually elicited many years after the maltreatment took place (Widom et al., 2007a), it is important to consider the potential impact of several biases – including forgetting (Hardt and Rutter, 2004), infantile amnesia (Howe and Courage, 1993), subsequent life-events (Tajima et al., 2004), and questionnaire or interview quality (Fergusson et al., 2000) – on the likelihood that early traumatic memories are recalled and disclosed. A more concerning issue is the potential influence that mood-congruent memory biases could exert on the salience of childhood memories. For example, adults with mental health problems such as depression might exhibit generalized difficulties with memory and attention compared to healthy adults (Brewin et al., 1993). Furthermore, depressed adults might remember more negative versus positive childhood memories, whereas mentally well adults might show the opposite trend (Widom and Morris, 1997). Such memory biases could confound or inflate the association between childhood maltreatment and adult psychopathology, which raises concerns over the validity of previous findings (Hardt and Rutter, 2004, Susser and Widom, 2012). Whilst, there is some experimental evidence of such ‘mood-congruent recall’ (Bower, 1981), most anecdotal evidence relates only to depression (Brewin et al., 1993). Moreover, Fergusson et al. (2011) showed – by fitting a structural equation model to repeated measures of mental health and retrospective self-reports of maltreatment – that report unreliability and recall bias had minimal influence on the variance of maltreatment self-reports over time. The potential influence of mood-congruent memory biases on retrospective self-reports of maltreatment therefore remains equivocal.

In theory, children could prospectively self-report on their maltreatment experiences. However, asking a young child questions about traumatic experiences could be distressing for the child, and therefore would usually be unethical and impractical in research studies. Official records, such as medical and social services files, are the most commonly used prospective measure of childhood maltreatment, and have typically been considered the gold-standard (Hardt and Rutter, 2004, Widom and Shepard, 1996) because such measures are comparatively independent (that is, independently substantiated by professionals). However, only a small proportion of maltreated children come to the attention of professionals, therefore exclusive reliance on official records drastically underestimates the prevalence of maltreatment (Gilbert et al., 2009, MacMillan et al., 2003).

Alternatively, prospective caregiver-reports (usually from the mother) can be used. Intuitively, we might expect parents to know the most information about whether their child has been maltreated. However, caregivers might clearly withhold information if they are the perpetrator or in a relationship with the perpetrator (Fisher et al., 2011). Parents could also simply be unaware of the maltreatment, which is particularly likely with sexual abuse which is usually secretive. Further, longitudinal studies can be subject to high rates of selective attrition during follow-up, where potentially those most likely to develop psychopathology drop out (Martin et al., 2016), thus limiting the generalizability of associations between prospectively measured maltreatment and adult psychopathology.

Considering the various potential biases of both retrospective self-reports and prospective reports of childhood maltreatment, further research into the validity and utility of both designs is needed. A small number of studies have addressed this by comparing prospective and retrospective measures of maltreatment from the same individuals. This provides the opportunity to assess between-method agreement and to contrast prospective and retrospective measures for their ability to statistically predict mental health outcomes. Between-method agreement has generally been low, ranging from slight (Henry et al., 1994, Johnson et al., 1999, Reuben et al., 2016) to fair (Patten et al., 2015, White et al., 2007). Retrospective self-reports (versus prospective measures) have tended to demonstrate stronger associations with adult psychopathology (Brown et al., 2005, Everson et al., 2008, Reuben et al., 2016, Tajima et al., 2004, Widom and Morris, 1997, Widom et al., 1999) – though the reverse has also been documented (Scott et al., 2012, Shaffer et al., 2008).

However, studies to date have typically obtained retrospective self-reports of maltreatment many years into adulthood, which increases the time in which forgetting and subsequent life-events could influence the recall of childhood experiences, and confound the association between maltreatment and adult psychopathology. Additionally, prospective measures of maltreatment have mostly been obtained from official records – which capture only a small proportion of cases. Further, studies have usually measured just one or two maltreatment types and psychiatric outcomes, which could partly explain the conflicting findings. For example, the relative predictive capacity of prospective reports versus retrospective self-reports could depend on which psychiatric outcome is examined.

The present study explores the validity and utility of retrospective self-reports versus prospective informant-reports of childhood maltreatment. Here we incorporate three novel design features. First, we obtained prospective informant-reports of maltreatment at several time-points during childhood via caregiver interviews (supplemented with researcher observations and clinicians’ reports where relevant), to foster trust between caregivers and research workers. Second, retrospective self-reports of maltreatment were obtained in early-adulthood (age 18) to reduce the time in which early childhood memories might be forgotten: at age-18, participants were reporting on events that happened between six and ∼18 years prior, rather than several decades earlier. Third, we examined various forms of maltreatment and several early-adult psychiatric problems to gauge whether findings might differ between maltreatment type and psychiatric outcome. We focussed specifically on early-adult psychiatric problems because the majority of individuals who will develop a mental health problem will have done so by this point (Kim-Cohen et al., 2003). With these measures, we asked: (1) Is there agreement between prospective informant-reports and retrospective self-reports of childhood maltreatment? (2) Do prospective reports and retrospective reports of maltreatment differ in their ability to predict early-adult psychiatric problems? And (3) Are prospective and retrospective reports of maltreatment independently associated with early-adult psychiatric problems? This step was conducted to test whether prospective informant-reports of maltreatment predicted early-adult psychiatric problems above and beyond the effect of retrospective self-reports (and vice versa).

## 2 Materials and methods

### 2.1 Study cohort

Participants were members of the Environmental Risk (E-Risk) Longitudinal Twin Study, which tracks the development of a nationally-representative birth cohort of 2232 British twin children. Full details about the sample are reported elsewhere (Moffitt and E-Risk Study Team, 2002) and in the Supplementary Materials. Briefly, the E-Risk sample was constructed in 1999–2000, when 1116 families (93% of those eligible) with same-sex 5-year-old twins participated in home-visit assessments. This sample comprised 56% monozygotic (MZ) and 44% dizygotic (DZ) twin pairs; sex was evenly distributed within zygosity (49% male). Families were recruited to represent the UK population of families with newborns in the 1990s, on the basis of residential location throughout England and Wales and mother's age.

Follow-up home-visits were conducted when children were aged 7, 10, 12 and 18 (participation rates were 98%, 96%, 96% and 93%, respectively). Home-visits at ages 5, 7, 10, and 12 years included assessments with participants as well as their mother (or primary caregiver); the home-visit at age 18 included interviews only with the participants. Each participant in a twin pair was privately assessed by a different interviewer. There were 2066 E-Risk participants who were assessed at age 18. The average age of the participants at the time of the assessment was 18.4 years (*SD* = 0.36); all interviews were conducted after the 18th birthday. Parents gave informed consent and twins gave assent between 5 and 12 years and then informed consent at age 18. The Joint South London and Maudsley and the Institute of Psychiatry Research Ethics Committee approved each phase of the study.

### 2.2 Measures

#### 2.2.1 Prospective informant-reports

Exposure to several types of maltreatment was assessed prospectively when the E-Risk participants were aged 5, 7, 10, and 12 (the age 5 assessment enquired about maltreatment since birth). Research workers visited the home in pairs, and were extensively trained to detect signs of abuse or neglect. During each visit, research workers interviewed the primary caregiver (usually the mother) using a structured interview about child harm, tested the children, and observed the family environment for evidence of neglect using the Home Observation for Measurement of the Environment (HOME) (Bradley and Caldwell, 1977). Specifically, caregivers were asked several questions about whether either of their twins had been intentionally harmed (physically or sexually) by an adult or had contact with welfare agencies. If caregivers endorsed a question, follow-up questions were asked and research workers made extensive notes on what had happened, and indicated whether physical and/or psychological harm had occurred. Under the UK Children Act, our responsibility was to secure intervention if maltreatment was current and ongoing. Such intervention on behalf of E-Risk families was carried out with parental cooperation in all but one case. No families left the study following intervention. An unusual feature of the E-Risk study's assessment is that we repeatedly interviewed mothers on four occasions over the years, which allowed them to build confidence in the research team. Also, we were able to reassure mothers that if harm to the child was ongoing and had to be reported by us, reporting would be managed through a trusted familiar professional, namely the family's registered General Practitioner. As the children grew older, some mothers who were initially reluctant to reveal abuse to us, divulged details of severe abuse at a later interview.

Comprehensive dossiers have been compiled for each child with cumulative information about exposure to physical abuse by an adult; sexual abuse; physical neglect; and emotional abuse/neglect. The dossiers comprised reports from caregivers of maltreatment, recorded narratives of the caregiver interviews, recorded debriefings with research workers who had coded any indication of abuse and neglect at any of the successive home visits, and information from clinicians whenever the study team made a child-protection referral. The dossiers were reviewed by two independent researchers and rated for the presence and severity (none/mild/severe) of each type of maltreatment. Inter-rater agreement between the coders exceeded 85% among the maltreatment cases, and discrepantly coded cases were resolved by consensus review. In the present study, each type of prospectively-reported maltreatment was dichotomized to represent none/mild (0) versus severe (1) maltreatment. Additional details about the prospective measure of childhood maltreatment have been reported previously (Danese et al., 2016, Fisher et al., 2015) and are provided in the Supplementary Materials.

Given the low prevalence of some specific forms of maltreatment (e.g., sexual abuse and physical neglect) we created an ‘any maltreatment’ composite by combining all forms of prospectively reported severe maltreatment. A severe rating of physical abuse, sexual abuse, physical neglect, and/or emotional abuse/neglect equated to a severe rating of ‘any maltreatment’. Additionally, given that adversities cluster (Kessler et al., 1997) and demonstrate cumulative associations with risk for psychopathology (Turner and Lloyd, 1995), we created a ‘multiple maltreatment’ variable by summing and categorizing all forms of severe maltreatment (range: 0 [no severe maltreatment]; 1 [one form of severe maltreatment]; 2 [two or more forms of severe maltreatment]).

#### 2.2.2 Retrospective self-reports

Maltreatment was measured retrospectively using the Childhood Trauma Questionnaire (CTQ; Bernstein and Fink, 1998) when E-Risk participants were aged 18. The CTQ is a 25-item questionnaire used for retrospective recall of five forms of maltreatment, and has high inter-rater reliability and construct and convergent validity (Fink et al., 1995). The CTQ is also one of the most commonly used retrospective measures of childhood maltreatment, thus increasing the comparability of the present study with previous and future research. Participants reported on their personal experiences of physical, sexual and emotional abuse, and physical and emotional neglect for the period before they were 12 years old (i.e., before entering secondary school). Almost all (99.5%; N = 2055) E-Risk participants who took part in the age-18 assessment completed the CTQ. This forms our analysis sample for the present study. Maltreatment scores were dichotomized following CTQ guidelines (Bernstein and Fink, 1998) to represent none/low (0) versus moderate/severe (1) maltreatment. To allow retrospective self-reports of maltreatment to be compared to prospective informant-reports, emotional abuse and emotional neglect were combined so that a moderate/severe score for emotional abuse and/or emotional neglect represented a moderate/severe score for ‘emotional abuse/neglect’. As with the prospective reports, we created any maltreatment and multiple maltreatment variables following the same procedure described above but using the retrospective self-reports of maltreatment.

#### 2.2.3 Early-adult psychiatric problems

During age-18 interviews, we assessed the presence of major depressive disorder (referred to as depression), generalized anxiety disorder (referred to as anxiety), self-injury (self-harm and/or suicide attempt), alcohol/cannabis dependence and conduct disorder according to Diagnostic and Statistical Manual of Mental Disorders (DSM-IV) criteria. The reporting period referred to the previous 12 months, although we used a 6-year reporting period for self-injury because suicide attempt is a rare event. Further, for the anxiety diagnosis, we did not require the 6-month symptom duration criterion because of the young age of our study sample. Assessments were conducted in face-to-face private interviews using the Diagnostic Interview Schedule (Robins et al., 1995). Conduct disorder was assessed as part of a computer-administered module, and was defined as a score of five or more out of 13 items (scored yes/no) assessing behavior such as bullying, physical cruelty, lying, and truancy, etc. The rates of psychiatric problems in this sample at age 18 were 20.1% (N = 412) for depression; 7.3% (N = 150) for anxiety; 14.2% (N = 291) for self-injury; 15.9% (N = 326) for alcohol/cannabis dependence; and 15.0% (N = 307) for conduct disorder. These rates are similar to those reported in other general population samples of a similar age (Costello et al., 2003, Hankin et al., 1998, Kidger et al., 2012, Merikangas et al., 2010).

### 2.3 Statistical analysis

All analyses were conducted in STATA 14.2 (StataCorp LP, USA), and proceeded in four steps. First, we calculated the agreement between prospective informant-reports and retrospective self-reports of any maltreatment and multiple maltreatment using Cohen's Kappa. We repeated this for the specific forms of childhood maltreatment. Second, we used logistic regression to calculate the associations of prospective informant-reports and retrospective self-reports of maltreatment with each of the early-adult psychiatric problems. Third, we repeated regression models with prospective informant-reports and retrospective self-reports of maltreatment entered simultaneously, to check whether both report types were independently associated with early-adult psychiatric problems. Fourth, because the prospective and retrospective variables entailed different thresholds, we conducted sensitivity analyses by repeating steps one to three using broader thresholds (no maltreatment versus any evidence of maltreatment). As this sample comprises twins, all regression analyses were adjusted for the non-independence of observations using the Huber/White variance estimator (Rogers, 1994).

## 3 Results

### 3.1 Is there agreement between prospective informant-reports and retrospective self-reports of childhood maltreatment?

Table 1 presents the prevalence of childhood maltreatment (any maltreatment, multiple maltreatment, and the specific forms of maltreatment) according to prospective informant-reports (obtained from caregiver reports, researchers' observations and clinicians’ reports between ages 5–12 years) and retrospective self-reports (reports by the participants themselves at age 18 of events before age 12), followed by concordance and agreement estimates. Comparable rates of maltreatment were identified by prospective (7.4%; N = 152) and retrospective (8.8%; N = 180) reports. Likewise, very similar rates of multiple maltreatment (2 or more forms) were identified by prospective (2.6%; N = 53) and retrospective (2.4%; N = 49) reports.

**Table 1:** Agreement between prospective informant-reports and retrospective self-reports of childhood maltreatment.

Maltreatment type	Prevalence of maltreatment		Concordance a		Agreement b	
	Prospective report	Retrospective report	Maltreatment present	Maltreatment absent	Absolute	Kappa
	%	%	%	%	%	К
	(n/N)	(n/N)	(n/N)	(n/N)		
Any maltreatment	7.4	8.8	27.63	92.8	87.9	0.188***
	(152/2055)	(180/2055)	(42/152)	(1765/1903)		

Multiple maltreatment c	2.6	2.4	26.42	98.3	92.2	0.205***
	(53/2055)	(49/2055)	(14/53)	(1967/2002)		

Specific forms of maltreatment						
Physical abuse	5.2	2.5	16.8	98.3	94.0	0.199***
	(107/2055)	(52/2055)	(18/107)	(1914/1948)		

Sexual abuse	0.7	1.1	40.0	99.2	98.7	0.310***
	(15/2055)	(23/2055)	(6/15)	(2023/2040)		

Physical neglect	1.7	1.3	11.8	98.9	97.4	0.118***
	(34/2055)	(27/2055)	(4/34)	(1998/2021)		

Emotional abuse/neglect	3.0	7.4	32.8	93.4	91.6	0.153***
	(61/2055)	(151/2055)	(20/61)	(1863/1994)		

Note: ***p < 0.001. Almost all (99.5%; N = 2055) E-Risk participants who took part in the age 18 assessment completed the Childhood Trauma Questionnaire. This forms our analysis sample.

a The concordance percentages (Maltreatment present, Maltreatment absent) were calculated using prospective reports of maltreatment as the reference (N). This is consistent with previous studies, and also takes into consideration the multiple-informant and multi-wave design of the prospective measure.

b Absolute agreement is the entire overlap between reports including instances where no maltreatment was reported. Kappa agreement takes into consideration chance overlap which can occur because the majority of children were not maltreated (and therefore most participants had a score of 0).

c Multiple forms of maltreatment (two or more forms of maltreatment which could include physical abuse, sexual abuse, physical neglect and/or emotional abuse/neglect) were reported prospectively/retrospectively. For the prevalence of maltreatment and concordance estimates, a binary multiple maltreatment variable was used (coded as 0/1 versus two or more forms of maltreatment). For the Kappa agreement analyses, an ordinal multiple maltreatment variable was used to capture the most information on agreement between reports, which ranged from 0 (no maltreatment) to 1 (one form of maltreatment) to 2+ (two or more forms of maltreatment). Weighted kappa was used for multiple maltreatment because the variable was on an ordinal scale.

However, there was low concordance between prospectively and retrospectively reported cases of maltreatment (Table 1). For example, only 27.6% of individuals with a prospective report of any maltreatment subsequently reported this maltreatment at age 18 (N = 42). Conversely, there was high concordance (92.8%; N = 1765) between prospective and retrospective reports of maltreatment in cases where maltreatment was prospectively reported as absent. Absolute between-method agreement was high owing to the low prevalence of maltreatment. In contrast, kappa agreement (К: agreement beyond that expected by chance) was slight (<0.2) for any maltreatment and fair (>0.2 and < 0.4) for multiple maltreatment (Sim and Wright, 2005), although both К estimates were statistically significant in our sample (p < 0.001).

Focussing on the specific forms of childhood maltreatment, low rates of sexual abuse and physical neglect and slightly higher rates of physical abuse and emotional abuse/neglect were identified, regardless of reporter. Kappa agreement between prospective and retrospective reports of the specific forms of maltreatment again ranged from slight to fair.

### 3.2 Do prospective reports and retrospective reports of maltreatment differ in their ability to predict early-adult psychiatric problems?

Table 2 shows that odds for all psychiatric problems were elevated (all ORs>1.0) among participants with reports of childhood maltreatment, regardless of report type, with many associations reaching a high level of statistical significance (p < 0.001). Retrospective (versus prospective) reports of maltreatment often produced stronger associations with psychiatric problems, particularly depression and self-injury. For example, the elevated odds for self-injury was over two-times greater for retrospective compared to prospective reports of maltreatment, and these effect size differences were statistically significant given the non-overlapping confidence intervals. Odds for psychiatric problems (notably depression and self-injury) were particularly high for individuals who retrospectively self-reported multiple (two or more) forms of childhood maltreatment. In contrast, the elevated odds for alcohol/cannabis dependence and conduct disorder did not substantially differ between retrospective and prospective reports. A similar pattern of associations was found when we examined the specific forms of maltreatment individually (see Supplementary Table 1).

**Table 2:** Associations of prospective informant-reports versus retrospective self-reports of childhood maltreatment with early-adult psychiatric problems.

Maltreatment type	Report type a	Association with early-adult psychiatric problems				
		Depression	Anxiety	Self-injury	Alcohol/cannabis dependence	Conduct disorder
		OR	OR	OR	OR	OR
		(95% CI)	(95% CI)	(95% CI)	(95% CI)	(95% CI)
Any maltreatment	Prospective report	2.37***	2.06*	2.42***	2.42***	3.23***
		(1.59, 3.54)	(1.18, 3.61)	(1.57, 3.79)	(1.62, 3.63)	(2.20, 4.73)
	Retrospective report	4.12***	3.08***	5.48***	3.30***	4.17***
		(3.00, 5.65)	(1.95, 4.88)	(3.88, 7.75)	(2.35, 4.62)	(2.96, 5.88)

Multiple maltreatment b	Prospective report					
	0	[reference]	[reference]	[reference]	[reference]	[reference]
	1	2.15**	1.87†	2.74***	2.63***	2.78***
		(1.32, 3.51)	(0.92, 3.84)	(1.69, 4.44)	(1.64, 4.22)	(1.71, 4.52)
	2+	2.83**	2.42*	1.93	2.07*	4.20***
		(1.49, 5.37)	(1.06, 5.50)	(0.83, 4.50)	(1.03, 4.17)	(2.40, 7.32)
	Retrospective report					
	0	[reference]	[reference]	[reference]	[reference]	[reference]
	1	3.10***	2.68**	3.88***	2.79***	4.34***
		(2.14, 4.50)	(1.53, 4.70)	(2.59, 5.81)	(1.88, 4.14)	(2.96, 6.36)
	2+	8.86***	4.26**	13.22***	4.99***	3.73***
		(4.84, 16.22)	(2.16, 8.42)	(6.96, 25.12)	(2.81, 8.85)	(1.95, 7.13)

Note: CI confidence interval, OR odds ratio. ***p < 0.001 **p < 0.01 * p < 0.05 †p < 0.1. Almost all (99.5%; N = 2055) E-Risk participants who took part in the age 18 assessment completed the Childhood Trauma Questionnaire. This forms our analysis sample. All analyses account for the non-independence of twin observations using the ‘cluster’ command in STATA.

a For report type, clear cells highlight the associations arising from prospective informant-reports of childhood maltreatment. Grey cells highlight the associations arising from retrospective self-reports of childhood maltreatment.

b Multiple forms of maltreatment (two or more forms of maltreatment which could include physical abuse, sexual abuse, physical neglect and/or emotional abuse/neglect) were reported prospectively/retrospectively. Regression analyses used the ordinal multiple maltreatment variable, which ranged from 0 (no maltreatment) to 1 (one form of maltreatment) to 2+ (two or more forms of maltreatment).

### 3.3 Are prospective reports and retrospective reports of maltreatment independently associated with early-adult psychiatric problems?

Prospective reports and retrospective reports of maltreatment were simultaneously entered into logistic regressions models to calculate their independent effects (Table 3). The associations arising from retrospective reports of any maltreatment and multiple maltreatment (two or more forms) mostly remained significant after considering the corresponding prospective report of maltreatment. That is, retrospective self-reports of maltreatment predicted early-adult psychopathology even in individuals without a corresponding prospective report of maltreatment. In contrast, many of the associations arising from prospective reports of maltreatment were attenuated to below conventional levels of significance after controlling for the corresponding retrospective report, particularly for multiple maltreatment which was no longer significantly associated with depression, anxiety, self-injury, or alcohol/cannabis dependence. That is, the association between prospective reports of maltreatment and psychiatric problems often did not hold among individuals who failed to also give a retrospective self-report of maltreatment. A similar pattern of attenuation (retrospective reports usually remaining robust and prospective reports often becoming non-significant) was found when we examined the specific forms of maltreatment individually (see Supplementary Table 2).

**Table 3:** Independent associations of prospective informant-reports versus retrospective self-reports of childhood maltreatment with early-adult psychiatric problems.

Maltreatment type	Report type a	Association with early-adult psychiatric problems b				
		Depression	Anxiety	Self-injury	Alcohol/cannabis dependence	Conduct disorder
		OR	OR	OR	OR	OR
		(95% CI)	(95% CI)	(95% CI)	(95% CI)	(95% CI)
Any maltreatment	Prospective report	1.81**	1.59	1.70*	1.92**	2.49***
		(1.19, 2.77)	(0.89, 2.85)	(1.04, 2.77)	(1.25, 2.95)	(1.62, 3.83)
	Retrospective report	3.74***	2.82***	5.02***	2.94***	3.57***
		(2.70, 5.17)	(1.76, 4.52)	(3.51, 7.17)	(2.06, 4.19)	(2.47, 5.17)

Multiple maltreatment c	Prospective report					
	0	[reference]	[reference]	[reference]	[reference]	[reference]
	1	1.89*	1.63	2.35**	2.35**	2.44**
		(1.15, 3.11)	(0.78, 3.42)	(1.40, 3.97)	(1.45, 3.81)	(1.45, 4.09)
	2+	1.44	1.41	0.63	1.15	2.78**
		(0.65, 3.20)	(0.58, 3.43)	(0.21, 1.92)	(0.51, 2.62)	(1.37, 5.62)
	Retrospective report					
	0	[reference]	[reference]	[reference]	[reference]	[reference]
	1	2.92***	2.53**	3.79***	2.62***	3.90***
		(2.00, 4.26)	(1.45, 4.40)	(2.51, 5.72)	(1.76, 3.92)	(2.60, 5.85)
	2+	7.95***	3.80**	14.94***	4.70***	2.69**
		(4.28, 14.75)	(1.79, 8.05)	(7.68, 29.04)	(2.50, 8.82)	(1.29, 5.59)

Note: CI confidence interval, OR odds ratio. ***p < 0.001 **p < 0.01 * p < 0.05. Almost all (99.5%; N = 2055) E-Risk participants who took part in the age 18 assessment completed the Childhood Trauma Questionnaire. This forms our analysis sample. All analyses account for the non-independence of twin observations using the ‘cluster’ command in STATA.

a For report type, clear cells highlight the associations arising from prospective informant-reports of childhood maltreatment. Grey cells highlight the associations arising from retrospective self-reports of childhood maltreatment.

b Associations of prospective informant-reports and retrospective self-reports of childhood maltreatment with early-adult psychiatric outcomes were modelled simultaneously. That is, prospective reports were adjusted for the corresponding retrospective self-report, and vice versa.

c Multiple forms of maltreatment (two or more forms of maltreatment which could include physical abuse, sexual abuse, physical neglect and/or emotional abuse/neglect) were reported prospectively/retrospectively. Regression analyses used an ordinal multiple maltreatment variable, which ranged from 0 (no maltreatment) to 1 (one form of maltreatment) to 2+ (two or more forms of maltreatment).

Repeating all of the above analyses with more broadly defined maltreatment variables (i.e., no maltreatment versus any evidence of maltreatment) produced comparable results (see Supplementary Results and Supplementary Tables 3, 4 and 5), suggesting that findings were not due to threshold differences between prospective and retrospective reports.

## 4 Discussion

The present study examined the validity and predictive utility of retrospective self-reports versus prospective informant-reports of childhood maltreatment. Our analyses revealed three main findings.

First, overall rates of childhood maltreatment, which closely match recent national and global estimates (Butchart et al., 2006, Gilbert et al., 2009, Radford et al., 2011, Radford et al., 2013), were similar between prospective and retrospective reports. However, agreement between prospective and retrospective reports of childhood maltreatment was only slight to fair (all K's ≤ 0.31). Between-method agreement was similar to that reported by Everson et al. (2008) in their comparison of official records and early-adolescent self-reports (all К’s ≤ 0.19); and slightly higher than recently reported by Reuben et al. (2016) in their comparison of researcher-reports and adult self-reports of maltreatment (all К’s ≤ 0.13). Our analyses revealed a tendency for participants with prospectively reported childhood maltreatment to underreport this maltreatment when interviewed at age 18. Conversely, most individuals who self-reported childhood maltreatment at age 18 did not have a corresponding prospective report during childhood. That is, despite using comprehensive and detailed measures of several forms of maltreatment that covered the same exposure period of birth to age 12, and despite obtaining self-reports of maltreatment at a relatively young age, our prospective and retrospective measures of childhood maltreatment captured two largely non-overlapping groups of maltreated individuals.

Second, participants who were maltreated during childhood were significantly more likely to have a range of psychiatric problems in early adulthood including depression, anxiety, self-injurious behavior, alcohol/cannabis dependence, and conduct disorder. These associations were apparent regardless of how maltreatment was measured, supporting the validity of the retrospective self-report measure used in this study. However, retrospective self-reports often demonstrated stronger associations with psychiatric problems, and particularly high odds for psychopathology were found among participants who self-reported multiple forms of maltreatment. These findings parallel results from previous studies (Everson et al., 2008, Tajima et al., 2004, Widom and Morris, 1997, Widom et al., 1999), and are consistent with recent evidence from the Dunedin Study in which adults' retrospective self-reports of adversity demonstrated stronger associations with subjective, self-reported health outcomes (Reuben et al., 2016). Several mechanisms might account for the differentially strong associations arising from retrospective self-reports. For example, prospective reports of maltreatment may have captured less severe cases of maltreatment due to parents' fear of disclosure. However, the prospective measure used in this study was carefully designed to foster trust between researchers and parents, and incorporated information from other informants. Alternatively, young adults with contemporaneous mental health problems might have remembered more negative versus positive childhood experiences because of their current mood. However, previous investigations using repeated measures of maltreatment and mental health indicate that such memory biases have minimal influence on the correlation between self-reports and psychopathology (Fergusson et al., 2011). It is also possible that individuals with current mental health problems were *more* accurate in retrieving memories of genuine maltreatment. Literature on ‘depressive realism’ (Ackermann and DeRubeis, 1991) suggests, for example, that depressed individuals are more accurate at perceiving reality as it is, whereas non-depressed individuals avoid unpleasant thoughts. However, another interpretation for the present findings is that young people were simply more knowledgeable about their childhood maltreatment experiences than their parents, making the self-report measure a better predictor of mental health.

Third, retrospective self-reports (versus prospective reports) of multiple maltreatment demonstrated particularly strong associations with psychiatric problems involving an affective component, including depression and self-injury (and to a lesser extent, anxiety). These differential associations were less apparent for alcohol/cannabis dependence and conduct disorder, which are often conceptualized as externalizing problems. Furthermore, the stronger associations between retrospective self-reports of multiple maltreatment and affective psychiatric problems were accentuated when retrospective and prospective reports were simultaneously modelled. That is, the associations arising from prospective reports often became non-significant or were substantially attenuated; whereas the associations arising from retrospective self-reports remained strong and significant. This suggests that that the link between maltreatment and affective forms of psychopathology in our sample was dependent on whether the childhood maltreatment was recalled in early-adulthood. In other words, maltreated individuals who themselves recalled being maltreated had a greater risk for affective problems than maltreated individuals who had forgotten (or chose not to disclose) this maltreatment.

### 4.1 Limitations

We acknowledge some limitations. First, the prevalence of both maltreatment and psychopathology in our sample was low. Analyses were adequately powered for our main research questions. However, only a small proportion of participants had double reports of any maltreatment (N = 42), which limited our ability to examine whether concordant reports of maltreatment provided additional information, such as indication of more severe maltreatment. Second, as with previous comparisons of prospective reports and retrospective self-reports, our analyses could not isolate the effects of timing from source. This is an unresolvable issue as it is not ethically appropriate in research studies to obtain self-reports prospectively from young children. However, the retrospective self-reports were obtained at a younger age than most previous studies, reducing the time in which childhood memories might be forgotten. Third, the CTQ is a questionnaire therefore our findings might not generalize to studies using different retrospective methods such as interviews. Nevertheless, given that the CTQ is one of the most commonly used retrospective maltreatment measures, our findings apply to a wide range of studies investigating psychological sequelae of childhood maltreatment. Finally, E-Risk is a twin sample, which could have a different risk profile to singleton samples. However, the prevalence of childhood maltreatment in our sample matches recent UK general population estimates (Radford et al., 2011, Radford et al., 2013). Additionally, the prevalence of psychopathology in twins and singletons has previously been shown not to differ (Gjone and Novik, 1995). Therefore, our findings should be generalizable to other cohorts covering similar time periods.

## 5 Conclusions

Given that prospective and retrospective reports yielded similar rates of maltreatment but captured largely non-overlapping groups, we suggest that both methodologies hold value in research studies. Prospective measures miss individuals whose maltreatment was not known or reported during childhood. Retrospective self-report measures miss individuals who have forgotten or choose not to disclose their childhood maltreatment experiences. Arguably the strongest approach, both to estimate the occurrence and the mental health consequences of maltreatment, is to use both prospective and retrospective measures in the same sample. A powerful method for studies benefitting from multiple sources of information on maltreatment could be to derive composite maltreatment variables from all available information. In our study, both prospective reports and retrospective reports of maltreatment were associated with early-adult psychiatric problems. This provides further evidence that previously documented associations between self-reported maltreatment and mental illness are not spurious, and indeed, retrospective self-reports are potentially a more useful indicator of clinical need. The growing support for specialist early-intervention facilities (Davidson et al., 2015) means that retrospective self-reports, via early-adulthood screening for childhood maltreatment, are an increasingly practical and useful measure of risk for psychopathology. Young adults who remember being maltreated could be a particularly high-risk group to target for early-intervention. However, it is unclear why the differentially strong associations of retrospective self-reports were mostly apparent for affective forms of psychopathology in our study. Future investigations using retrospective self-reports of childhood maltreatment may need to consider current mood state to account for potential memory biases.

## Financial Support

The E-Risk Study is funded by the United Kingdom Medical Research Council (UKMRC grant number: G1002190); Economic and Social Research Council (ESRC grant number: ES/M010309/1); National Institute of Child Health and Development (NICHD grant number: HD077482); Avielle Foundation; and by the Jacobs Foundation. JBN and JRB are supported by ESRC multidisciplinary studentships. HLF is supported by an MQ Fellows Award (grant number: MQ14F40). LA is the Mental Health Leadership Fellow for the UK ESRC.

## Conflict of interest

None.

## Appendix A Supplementary data

Newbury et al. Supplementary Materials Newbury et al. Supplementary Materials
