# Supplementary material for: Measuring childhood maltreatment to predict early-adult psychopathology: Comparison of prospective informant-reports and retrospective self-reports
Source: J Psychiatr Res. 2018 Jan;96:57–64. doi: 10.1016/j.jpsychires.2017.09.020 (PMC5725307; doi:10.1016/j.jpsychires.2017.09.020)
Supplement: Newbury et al. Supplementary Materials [file mmc1.docx]

**Supplementary Materials**

**Methods**

***Study Cohort***

Participants were members of the Environmental Risk (E-Risk) Longitudinal Twin Study, which tracks the development of a nationally-representative birth cohort of 2,232 British twin children. The sample was drawn from a larger birth register of twins born in England and Wales in 1994-1995 (Trouton *et al.* 2002). Full details about the sample are reported elsewhere (Moffitt & E-Risk Study Team, 2002). Briefly, the E-Risk sample was constructed in 1999-2000, when 1,116 families (93% of those eligible) with same-sex 5-year-old twins participated in home-visit assessments. This sample comprised 56% monozygotic (MZ) and 44% dizygotic (DZ) twin pairs; sex was evenly distributed within zygosity (49% male). Families were recruited to represent the UK population of families with newborns in the 1990s, on the basis of residential location throughout England and Wales and mother’s age. Teenaged mothers with twins were over-selected to replace high-risk families who were selectively lost to the register through non-response. Older mothers having twins via assisted reproduction were under-selected to avoid an excess of well-educated older mothers.

Follow-up home-visits were conducted when children were aged 7, 10, 12 and 18 (participation rates were 98%, 96%, 96% and 93%, respectively). Home-visits at ages 5, 7, 10, and 12 years included assessments with participants as well as their mother (or primary caregiver); the home-visit at age 18 included interviews only with the participants. Each participant in a twin pair was assessed by a different interviewer. There were 2,066 E-Risk participants who were assessed at age 18. The average age of the participants at the time of the assessment was 18.4 years (*SD*=0.36); all interviews were conducted after the 18^th^ birthday. There were no differences between those who did and did not take part at age 18 in terms of socioeconomic status (SES) assessed when the cohort was initially defined (χ^2^=0.86, *p*=0.65), age-5 IQ scores (*t*=0.98, *p*=0.33), or age-5 internalizing or externalizing behavior problems (*t*=0.40, *p*=0.69 and *t*=0.41, *p*=0.68, respectively). E-Risk families are representative of UK households across the spectrum of neighborhood-level deprivation: 25.6% of E-Risk families live in “wealthy achiever” neighborhoods compared to 25.3% of households nation-wide; 5.3% vs 11.6% live in “urban prosperity” neighborhoods; 29.6% vs 26.9% live in “comfortably off” neighborhoods; 13.4% vs 13.9% live in “moderate means” neighborhoods; and 26.1% vs 20.7% live in “hard-pressed” neighborhoods (CACI Information Services, 2006; Caspi et al., 2000). Parents gave informed consent and twins gave assent between 5-12 years and then informed consent at age 18. The Joint South London and Maudsley and the Institute of Psychiatry Research Ethics Committee approved each phase of the study.

**Prospective measures of childhood maltreatment**

Exposure to several types of maltreatment was assessed repeatedly when the children were 5, 7, 10, and 12 years of age and dossiers have been compiled for each child with cumulative information about exposure to physical abuse by an adult; sexual abuse; physical neglect; and emotional abuse/neglect. The E-Risk team has previously reported evidence on the reliability and validity of the measures of physical maltreatment and sexual abuse ([Jaffee, Caspi, Moffitt, & Taylor, 2004](#_ENREF_25)), emotional abuse/neglect (Danese et al., 2017), and physical neglect (Fisher et *al*., 2015). All the component measures are outlined briefly below.

*Physical and sexual harm by an adult.* We assessed childhood physical and sexual harm in the E-Risk Study using an approach that resembles the process undertaken by child protection agencies. Essentially this is a two-stage process. In child protection, professionals such as teachers working with children typically raise concerns if they observe signs or symptoms or if they become aware of risk that children are victims of violence. When concerns are raised, child protection officers then review the concerns and evaluated them in the context of information previously gathered on that child or family in order to determine the likelihood that abuse has taken place. In the E-Risk Study, research workers visited the home in pairs, and were extensively trained to detect signs of abuse or neglect. Each time the two research workers visited a home, they interviewed the mother using a structured interview about child harm, tested the children, and observed the family environment using the Home Observation for Measurement of the Environment (HOME) (Bradley and Caldwell, 1977). If either research worker had any concerns, they flagged up the case for review. Immediately after each home visit, a review was performed if a family was flagged. In addition, at each wave, any family who had been flagged on a prior wave of the study was automatically reviewed again. The reviews were performed independently by at least 2 clinical psychologists or psychiatrists, and were based on comprehensive dossiers compiled across multiple home visits for each study member during the course of the ongoing longitudinal study.

An unusual feature of the E-Risk study’s assessment is that we repeatedly interviewed mothers on four occasions over the years, which allowed them to build confidence in the research team. Also, we were able to reassure mothers that if harm to the child was ongoing and had to be reported by us, reporting would be managed through a trusted familiar professional, namely the family’s registered GP. As the children grew older, some mothers who were initially reluctant to reveal abuse to us, divulged details of severe abuse at a later interview.

At age 5, assessments were based on the standardised clinical protocol from the MultiSite Child Development Project (Dodge, Bates, & Pettitt, 1990; Lansford *et al*., 2002). At ages 7, 10, and 12 this interview was modified to expand its coverage of contexts for child harm. Interviews were designed to enhance mothers’ comfort with reporting valid child maltreatment information, while also meeting researchers’ responsibilities for referral under the UK Children Act. Specifically, mothers were asked whether either of their twins had been intentionally harmed (physically or sexually) by an adult or had contact with welfare agencies. If caregivers endorsed a question, research workers made extensive notes on what had happened, and indicated whether physical and/or psychological harm had occurred. Under the UK Children Act, our responsibility was to secure intervention if maltreatment was current and ongoing. Such intervention on behalf of E-Risk families was carried out with parental cooperation in all but one case. No families left the study following intervention.

Over the years of data collection, the study developed a cumulative profile for each child, comprising the caregiver reports, recorded debriefings with research workers who had coded any indication of maltreatment at any of the successive home visits, recorded narratives of the successive caregiver interviews, and information from clinicians whenever the Study team made a child-protection referral. Each time we visited a home, the research workers flagged concerns, and if there was sufficient evidence to code definite harm then we did so. If evidence only met the level of probable harm, we kept an “ongoing concern list” and if, at a later wave, there was continued evidence of probable harm, or new evidence, the code was upgraded to definite harm. The profiles were reviewed at the end of the age-12 phase by at least two clinical psychologists or psychiatrists. Initial inter-rater agreement between the coders was 90% in cases for whom maltreatment was identified (100% for cases of sexual abuse), and discrepantly coded cases were resolved by consensus review. These were coded as: 0 = no physical harm at any age; 1 = probable physical harm at any age; and 2 = definite physical harm at any age. When limited to the 2055 participants with CTQ data, there were 12.5% of children coded as probably being exposed to physical harm and 7.4% as definitely physically harmed by 12 years of age. There were 1.6% of the children coded as probably or definitely being exposed to sexual abuse.

*Physical neglect.* The cumulative observations of the physical state of the home environment documented by the research workers during home visits to the twins at ages 5, 7, 10 and 12 were reviewed by two raters for evidence of physical neglect. This was defined as any sign that the caretaker was not providing a safe, sanitary, or healthy environment for the child. This included the child not having proper clothing or food, as well as grossly unsanitary home environments (However, this did not include a family living in a crime-ridden neighborhood for economic reasons). Inter-rater agreement between the coders exceeded 85%, and discrepantly coded cases were resolved by consensus review. When limited to the 2055 participants with CTQ data, there 7.3% children with indication of minor physical neglect (coded 1), and where there were 1.7% children with indication of more severe physical neglect (coded 2).

*Emotional abuse and neglect.* These forms of maltreatment were coded from research workers’ narratives of the home visits at ages 5, 7, 10, and 12. We coded quite severe examples of parental behavior observed. For example, a mother who had schizophrenia screamed and swore at the children throughout the home visit. As another example, a father who was drunk during the home visit repeatedly spoke abusively to the children in front of the research workers. We found that coders could not empirically separate emotional abuse and emotional neglect in a reliable way and thus such experiences were coded together as emotional abuse/neglect. Inter-rater agreement between the coders exceeded 85% for cases with emotional abuse and neglect, and discrepant cases were resolved by consensus review. Children with no evidence of emotional abuse/neglect were coded as 0 (88.5%), those where there was some indication of emotionally inappropriate/potentially abusive or neglectful behavior were coded as 1 (8.5%), and where there was evidence of severe emotional abuse/neglect the children were coded as 2 (3.0%).

**Results**

*Sensitivity check: are findings consistent when maltreatment is defined more broadly?*

Supplementary Tables 3, 4 and 5 present results repeated with more broadly defined maltreatment variables (i.e., no maltreatment versus any evidence of maltreatment). Despite the higher overall rates of maltreatment, kappa agreement was similar to that obtained using the original severity thresholds. Further, retrospective self-reports still identified only a fifth to a half of prospectively reported cases of maltreatment (Supplementary Table 3). In addition, retrospective self-reports (versus prospective reports) still produced stronger associations with early-adult psychiatric problems, though the effect size differences were less marked (Supplementary Table 4). These effect size differences again became more apparent when retrospective self-reports and prospective reports were simultaneously modelled. That is, prospective reports were often attenuated to below conventional levels of significance after considering corresponding retrospective self-reports (particularly for multiple maltreatment), whereas retrospective self-reports unanimously remained robust to adjustment for corresponding prospective reports (Supplementary Table 5).

**References**

Bradley, R., & Caldwell, B., 1977. Home observation for measurement of the environment: a validation study of screening efficiency. **Am. J. Ment. Defic**. 81 (5), 417-420.

CACI Information Services, 2006. ACORN user guide. CACI, London, UK.

Caspi, A., Taylor, A., Moffitt, T.E., & Plomin, R., 2000. Neighborhood deprivation affects children's mental health: Environmental risks identified in a genetic design. Psychol. Sci. 11 (4), 338-342.

Danese, A., Moffitt, T.E., Arseneault, L., Bleiberg, B.A., Dinardo, P.B., Gandelman, S.B., Houts, R., Ambler, A., Fisher, H.L., & Poulton, R., 2017. The origins of cognitive deficits in victimized children: implications for neuroscientists and clinicians. Am. J. Psychiatry 174 (4), 349-361.

Dodge, K.A., Bates, J.E., & Pettit, G. S., 1990. Mechanisms in the cycle of violence. Science 250, 1678-1683.

Fisher, H.L., Caspi, A., Moffitt, T.E., Wertz, J., Gray, R., Newbury, J., Ambler, A., Zavos, H., Danese, A., Mill, J., Odgers, C.L., Pariante, C., Wong, C., C, Y., & Arseneault, L., 2015. Measuring adolescents' exposure to victimization: The Environmental Risk (E-Risk) Longitudinal Twin Study. Dev. Psychopathol. 27 (4pt2), 1399-1416.

Jaffee, S.R., Caspi, A., Moffitt, T.E., & Taylor, A., 2004. Physical maltreatment victim to antisocial child: evidence of an environmentally mediated process. J. Abnorm. Psychol. 113 (1), 44-55.

Lansford, J.E., Dodge, K.A., Pettit, G.S., Bates, J.E., Crozier, J., & Kaplow, J., 2002. Long-term effects of early child physical maltreatment on psychological, behavioral, and academic problems in adolescence: a 12-year prospective study. Arch. Pediatr. Adolesc. Med. 156 (8), 824-830.

Moffitt, T.E., & E-Risk Study Team, 2002. Teen-aged mothers in contemporary Britain. J. Child Psychol. Psychiatry 43(6), 727-742.

Trouton, A., Spinath, F.M., & Plomin, R., 2002. Twins early development study (TEDS): a multivariate, longitudinal genetic investigation of language, cognition and behavior problems in childhood. Twin Res. 5(5), 444-448.

**Supplementary Table 1** *Associations of prospective informant-reports versus retrospective self-reports of specific forms of childhood maltreatment with early-adult psychiatric problems.*

| **Specific forms of maltreatment** | **Report type** ^a^ | **Association with adolescent mental health problems** | | | | |
| --- | --- | --- | --- | --- | --- | --- |
|  |  | Depression | Anxiety | Self-injury | Alcohol/cannabis dependence | Conduct disorder |
|  |  | OR  (95% CI) | OR  (95% CI) | OR  (95% CI) | OR  (95% CI) | OR  (95% CI) |
|  |  |  |  |  |  |  |
| Physical abuse | Prospective report | 2.12**  (1.31, 3.43) | 2.37**  (1.29, 4.36) | 2.27**  (1.34, 3.83) | 2.28**  (1.43, 3.65) | 3.74***  (2.45, 5.70) |
|  | Retrospective report | 5.32***  (2.99, 9.49) | 3.59***  (1.75, 7.36) | 8.37***  (4.48, 15.65) | 4.82***  (2.77, 8.40) | 5.18***  (2.84, 9.45) |
|  |  |  |  |  |  |  |
| Sexual abuse | Prospective report | 11.22***  (4.06, 31.03) | 1.96  (0.42, 9.21) | 5.41**  (1.62, 18.00) | 1.94  (0.62, 6.01) | 2.07  (0.67, 6.44) |
|  | Retrospective report | 6.37***  (2.77, 14.64) | 3.60*  (1.28, 10.14) | 11.92***  (4.85, 29.30) | 1.89  (0.65, 5.45) | 1.19  (0.42, 3.41) |
|  |  |  |  |  |  |  |
| Physical neglect | Prospective report | 2.21†  (0.98, 4.94) | 1.71  (0.48, 6.01) | 1.89  (0.73, 4.88) | 2.59*  (1.20, 5.60) | 2.77**  (1.41, 5.45) |
|  | Retrospective report | 4.40***  (2.17, 8.95) | 2.23  (0.84, 5.94) | 10.88***  (4.42, 26.77) | 2.70*  (1.25, 5.80) | 2.11  (0.89, 4.98) |
|  |  |  |  |  |  |  |
| Emotional abuse/neglect | Prospective report | 2.88**  (1.54, 5.36) | 2.59*  (1.16, 5.78) | 1.85  (0.86, 3.95) | 2.29*  (1.16, 4.50) | 2.88**  (1.57, 5.31) |
|  | Retrospective report | 5.01***  (3.54, 7.09) | 3.52***  (2.21, 5.60) | 5.79***  (4.03, 8.32) | 3.68***  (2.59, 5.24) | 4.03***  (2.81, 5.78) |
|  |  |  |  |  |  |  |

Note: CI confidence interval, OR odds ratio. *** p<0.001 ** p<0.01 * p<0.05 † p<0.1. Almost all (99.5%; N=2055) E-Risk participants who took part in the age-18 assessment completed the Childhood Trauma Questionnaire. This forms our analysis sample. ^a^ For report type, clear cells highlight the associations arising from prospective informant-reports of childhood maltreatment. Grey cells highlight the associations arising from retrospective self-reports of childhood maltreatment. All analyses account for the non-independence of twin observations using the ‘cluster’ command in STATA.

**Supplementary Table 2** *Independent associations of prospective informant-reports versus retrospective self-reports of specific forms of childhood maltreatment with early-adult psychiatric problems.*

| **Specific forms of maltreatment** | **Report type** ^a^ | **Association with adolescent mental health problems** | | | | |
| --- | --- | --- | --- | --- | --- | --- |
|  |  | Depression | Anxiety | Self-injury | Alcohol/cannabis dependence | Conduct disorder |
|  |  | OR  (95% CI) | OR  (95% CI) | OR  (95% CI) | OR  (95% CI) | OR  (95% CI) |
|  |  |  |  |  |  |  |
| Physical abuse | Prospective report | 1.65†  (0.96, 2.82) | 1.93†  (0.96, 3.87) | 1.57  (0.83, 2.94) | 1.79*  (1.05, 3.05) | 3.05***  (1.89, 4.92) |
|  | Retrospective report | 4.61***  (2.49, 8.55) | 2.89*  (1.26, 6.60) | 7.36***  (3.81, 14.21) | 4.06***  (2.21, 7.46) | 3.76***  (1.85, 7.65) |
|  |  |  |  |  |  |  |
| Sexual abuse | Prospective report | 7.18**  (1.94, 26.63) | 1.04  (0.17, 6.51) | 2.24  (0.39, 13.06) | 1.56  (0.38, 6.47) | 2.11  (0.60, 7.48) |
|  | Retrospective report | 4.35**  (1.60, 11.82) | 3.57*  (1.12, 11.35) | 9.92***  (3.84, 25.59) | 1.67  (0.46, 6.04) | 0.95  (0.28, 3.24) |
|  |  |  |  |  |  |  |
| Physical neglect | Prospective report | 1.89  (0.77, 4.65) | 1.55  (0.43, 5.56) | 1.36  (0.36, 5.09) | 2.34*  (1.03, 5.31) | 2.59**  (1.28, 5.25) |
|  | Retrospective report | 4.06***  (1.90, 8.70) | 2.09  (0.77, 5.67) | 10.46***  (4.19, 26.14) | 2.39*  (1.05, 5.45) | 1.82  (0.72, 4.55) |
|  |  |  |  |  |  |  |
| Emotional abuse/neglect | Prospective report | 1.95†  (0.99, 3.83) | 1.78  (0.79, 4.04) | 1.05  (0.48, 2.30) | 1.60  (0.77, 3.34) | 2.01†  (0.98, 4.11) |
|  | Retrospective report | 4.68***  (3.28, 6.68) | 3.27***  (2.05, 5.24) | 5.76  (4.00, 8.29) | 3.49***  (2.43, 5.02) | 3.73***  (2.56, 5.42) |
|  |  |  |  |  |  |  |

Note: CI confidence interval, OR odds ratio. *** p<0.001 ** p<0.01 * p<0.05 † p<0.1. Almost all (99.5%; N=2055) E-Risk participants who took part in the age-18 assessment completed the Childhood Trauma Questionnaire. This forms our analysis sample. ^a^ For report type, clear cells highlight the associations arising from prospective informant-reports of childhood maltreatment. Grey cells highlight the associations arising from retrospective self-reports of childhood maltreatment. ^b^ Associations of prospective informant-reports and retrospective self-reports of childhood maltreatment with early-adult psychiatric outcomes were modelled simultaneously. That is, prospective reports were adjusted for the corresponding retrospective self-report, and vice versa. All analyses account for the non-independence of twin observations using the ‘cluster’ command in STATA.

**Supplementary Table 3** *Agreement between prospective informant-reports and retrospective self-reports of childhood maltreatment using broadly defined maltreatment variables.*

| **Maltreatment type** | **Prevalence of maltreatment** | | **Concordance** ^a^ | | **Agreement** ^b^ | |
| --- | --- | --- | --- | --- | --- | --- |
|  | Prospective report | Retrospective report | Maltreatment present | Maltreatment absent | Absolute | Kappa |
|  | % | % | % | % | % | К |
|  | (n/N) | (n/N) | (n/N) | (n/N) |  |  |
|  |  |  |  |  |  |  |
| Any maltreatment | 26.0 | 40.2 | 52.6 | 64.2 | 61.2 | 0.143*** |
|  | (534/2055) | (826/2055) | (281/534) | (976/1521) |  |  |
|  |  |  |  |  |  |  |
| Multiple maltreatment ^c^ | 12.1 | 10.1 | 29.0 | 92.5 | 73.0 | 0.170*** |
|  | (248/2055) | (208/2055) | (72/248) | (1671/1807) |  |  |
|  |  |  |  |  |  |  |
| Specific forms of maltreatment |  |  |  |  |  |  |
|  |  |  |  |  |  |  |
|  |  |  |  |  |  |  |
| Physical abuse | 19.9 | 8.7 | 19.6 | 94.0 | 79.2 | 0.172*** |
|  | (409/2055) | (179/2055) | (80/409) | (1547/1646) |  |  |
|  |  |  |  |  |  |  |
| Sexual abuse | 1.6 | 1.5 | 21.9 | 98.9 | 97.7 | 0.214*** |
|  | (32/2055) | (30/2055) | (7/32) | (2000/2023) |  |  |
|  |  |  |  |  |  |  |
| Physical neglect | 8.9 | 6.7 | 19.1 | 94.5 | 87.8 | 0.153*** |
|  | (183/2055) | (138/2055) | (35/183) | (1769/1872) |  |  |
|  |  |  |  |  |  |  |
| Emotional abuse/neglect | 11.5 | 19.8 | 55.3 | 65.4 | 64.2 | 0.105*** |
|  | (237/2055) | (407/2055) | (131/237) | (1188/1818) |  |  |
|  |  |  |  |  |  |  |

Note: *** p<0.001. Almost all (99.5%; N=2055) E-Risk participants who took part in the age-18 assessment completed the Childhood Trauma Questionnaire. This forms our analysis sample**.** ^a^ The concordance percentages (Maltreatment present, Maltreatment absent) were calculated using prospective reports of maltreatment as the reference (N). This is consistent with previous studies, and also takes into consideration the multiple-informant and multi-wave design of the prospective measure. ^b^ Absolute agreement is the entire overlap between reports including instances where no maltreatment was reported. Kappa agreement takes into consideration chance overlap which can occur because the majority of children were not maltreated (and therefore most participants had a score of 0). ^c^ Multiple forms of maltreatment (two or more forms of maltreatment which could include physical abuse, sexual abuse, physical neglect and/or emotional abuse/neglect) were reported prospectively/retrospectively. For the prevalence of maltreatment and concordance estimates, a binary multiple maltreatment variable was used (coded as 0/1 versus two or more forms of maltreatment). For the Kappa agreement analyses, an ordinal multiple maltreatment variable was used to capture the most information on agreement between reports, which ranged from 0 (no maltreatment) to 1 (one form of maltreatment) to 2+ (two or more forms of maltreatment). Weighted kappa was used for multiple maltreatment because the variable was on an ordinal scale.

**Supplementary Table 4** *Associations of prospective informant-reports versus retrospective self-reports of childhood maltreatment with early-adult psychiatric problems using broadly defined maltreatment variables.*

| **Maltreatment type** | **Report type** ^a^ | **Association with early-adult psychiatric problems** | | | | |
| --- | --- | --- | --- | --- | --- | --- |
|  |  | Depression | Anxiety | Self-injury | Alcohol/cannabis dependence | Conduct disorder |
|  |  | OR  (95% CI) | OR  (95% CI) | OR  (95% CI) | OR  (95% CI) | OR  (95% CI) |
|  |  |  |  |  |  |  |
| Any maltreatment | Prospective report | 1.56***  (1.22, 2.01) | 1.28  (0.90, 1.90) | 1.76***  (1.32, 2.34) | 1.62***  (1.24, 2.12) | 2.15***  (1.63, 2.84) |
|  | Retrospective report | 1.87***  (1.48, 2.36) | 1.83**  (1.30, 2.59) | 2.90***  (2.23, 3.77) | 2.40***  (1.88, 3.06) | 2.93***  (2.26, 3.79) |
|  |  |  |  |  |  |  |
| Multiple maltreatment ^b^ | Prospective report  0 | [reference] | [reference] | [reference] | [reference] | [reference] |
|  | 1 | 1.62**  (1.19, 2.20) | 1.59†  (0.99, 2.54) | 1.63**  (1.16, 2.29) | 1.79***  (1.29, 2.49) | 1.77**  (1.23, 2.55) |
|  | 2+ | 1.51*  (1.08, 2.11) | 0.94  (0.51, 1.70) | 1.90**  (1.29, 2.81) | 1.43†  (0.98, 2.07) | 2.62***  (1.85, 3.71) |
|  | Retrospective report  0 | [reference] | [reference] | [reference] | [reference] | [reference] |
|  | 1 | 1.55**  (1.20, 1.99) | 1.61*  (1.10, 2.36) | 2.13***  (1.60, 2.83) | 1.80***  (1.37, 2.35) | 2.18***  (1.64, 2.89) |
|  | 2+ | 3.05***  (2.20, 4.24) | 2.52***  (1.57, 4.03) | 5.91***  (4.11, 8.51) | 4.72***  (3.33, 6.67) | 5.91***  (4.14, 8.45) |
|  |  |  |  |  |  |  |

Note: CI confidence interval, OR odds ratio. *** p<0.001 ** p<0.01 * p<0.05 † p<0.1. Almost all (99.5%; N=2055) E-Risk participants who took part in the age-18 assessment completed the Childhood Trauma Questionnaire. This forms our analysis sample**.** ^a^ For report type, clear cells highlight the associations arising from prospective informant-reports of childhood maltreatment. Grey cells highlight the associations arising from retrospective self-reports of childhood maltreatment. ^b^ Multiple forms of maltreatment (two or more forms of maltreatment which could include physical abuse, sexual abuse, physical neglect and/or emotional abuse/neglect) were reported prospectively/retrospectively. Regression analyses used the ordinal multiple maltreatment variable, which ranged from 0 (no maltreatment) to 1 (one form of maltreatment) to 2+ (two or more forms of maltreatment). All analyses account for the non-independence of twin observations using the ‘cluster’ command in STATA.

**Supplementary Table 5** *Independent associations of prospective informant-reports versus retrospective self-reports of childhood maltreatment with early-adult psychiatric problems using broadly defined maltreatment variables.*

| **Maltreatment type** | **Report type** ^a^ | **Association with early-adult psychiatric problems** ^b^ | | | | |
| --- | --- | --- | --- | --- | --- | --- |
|  |  | Depression | Anxiety | Self-injury | Alcohol/cannabis dependence | Conduct disorder |
|  |  | OR  (95% CI) | OR  (95% CI) | OR  (95% CI) | OR  (95% CI) | OR  (95% CI) |
|  |  |  |  |  |  |  |
| Any maltreatment | Prospective report | 1.43**  (1.11, 1.83) | 1.16  (0.79, 1.71) | 1.50**  (1.12, 2.02) | 1.42*  (1.08, 1.87) | 1.86***  (1.40, 2.46) |
|  | Retrospective report | 1.78***  (1.41, 2.25) | 1.79**  (1.28, 2.52) | 2.75***  (2.11, 3.59) | 2.29***  (1.79, 2.92) | 2.71***  (2.08, 3.51) |
|  |  |  |  |  |  |  |
| Multiple maltreatment ^c^ | Prospective report  0 | [reference] | [reference] | [reference] | [reference] | [reference] |
|  | 1 | 1.48*  (1.09, 2.02) | 1.45  (0.91, 2.31) | 1.40†  (0.98, 1.99) | 1.57*  (1.11, 2.22) | 1.54*  (1.05, 2.25) |
|  | 2+ | 1.16  (0.82, 1.63) | 0.62  (0.40, 1.30) | 0.22  (0.80, 1.87) | 0.94  (0.63, 1.41) | 1.77**  (1.22, 2.58) |
|  | Retrospective report  0 | [reference] | [reference] | [reference] | [reference] | [reference] |
|  | 1 | 1.52**  (1.18, 1.97) | 1.62*  (1.11, 2.36) | 2.09***  (1.57, 2.79) | 1.78***  (1.36, 2.34) | 2.10***  (1.58, 2.79) |
|  | 2+ | 2.88***  (2.06, 4.03) | 2.65***  (1.66, 4.24) | 5.51***  (3.74, 8.12) | 4.69***  (3.25, 6.77) | 4.97***  (3.40, 7.26) |
|  |  |  |  |  |  |  |

Note: CI confidence interval, OR odds ratio. *** p<0.001 ** p<0.01 * p<0.05. Almost all (99.5%; N=2055) E-Risk participants who took part in the age-18 assessment completed the Childhood Trauma Questionnaire. This forms our analysis sample**.** ^a^ For report type, clear cells highlight the associations arising from prospective informant-reports of childhood maltreatment. Grey cells highlight the associations arising from retrospective self-reports of childhood maltreatment. ^b^ Associations of prospective informant-reports and retrospective self-reports of childhood maltreatment with early-adult psychiatric outcomes were modelled simultaneously. That is, prospective reports were adjusted for the corresponding retrospective self-report, and vice versa. ^c^ Multiple forms of maltreatment (two or more forms of maltreatment which could include physical abuse, sexual abuse, physical neglect and/or emotional abuse/neglect) were reported prospectively/retrospectively. Regression analyses used an ordinal multiple maltreatment variable, which ranged from 0 (no maltreatment) to 1 (one form of maltreatment) to 2+ (two or more forms of maltreatment). All analyses account for the non-independence of twin observations using the ‘cluster’ command in STATA.
